# Recurrent ZFX mutations in human sporadic parathyroid adenomas

**DOI:** 10.18632/oncoscience.116

**Published:** 2014-05-06

**Authors:** Chen-Pang Soong, Andrew Arnold

**Affiliations:** ^1^ University of Connecticut School of Medicine, Center for Molecular Medicine

**Keywords:** oncogene, primary hyperparathyroidism, zinc finger, parathyroid hormone

## Abstract

The molecular abnormalities leading to sporadic parathyroid adenomas, a common type of human endocrine neoplasm, are heterogeneous and incompletely understood. Using whole exome and direct sequencing of parathyroid adenoma DNA samples, we identified recurrent somatic mutations in the *ZFX* gene. *ZFX* is a member of Krueppel C2H2 type zinc finger protein family, was initially described as a homolog of *ZFY*, and has been implicated as a transcription factor regulating embryonic stem cell renewal. The *ZFX* mutations we identified were strikingly specific, focused in each tumor on one encoded residue in a hotspot of two consecutive highly conserved arginine residues (R786/787; arginine to glutamine, threonine or leucine) in a zinc finger domain near the C-terminus of the protein. The intragenic specificity of these recurrently selected mutations, their confirmed expression within the tumors, the absence of loss of heterozygosity, and the absence of these mutations among over 4000 *ZFX* alleles in the dbSNP137 database, strongly suggest a novel role for *ZFX* as a human proto-oncogene. Further, these observations highlight the mutated zinc-finger domain as a new focal point for understanding *ZFX*'s normal and tumorigenic functions, and for development of molecularly-targeted therapeutics.

## INTRODUCTION

Sporadic parathyroid adenomas are the most common cause of primary hyperparathyroidism, a common endocrine disorder that affects both genders and all ages, and is most notably found in 2.1% of post-menopausal women [1]. Parathyroid adenomas release excessive quantities of parathyroid hormone (PTH), which can lead to hypercalcemia, osteoporosis, skeletal fractures, kidney stones, and nephrocalcinosis.

Previous investigations of the molecular pathogenesis of sporadic parathyroid adenomas identified recurrent, clonally selected driver mutations in the cyclin D1 (*CCND1*) proto-oncogene and *MEN1* tumor suppressor gene, and a combination of rare germline variants and somatic alterations in several cyclin-dependent kinase inhibitor genes [2-6]. However, these derangements occur, collectively, in a minority of these tumors [2, 3, 7], and even when present are likely to cooperate with other driver lesions; thus multiple additional targets for tumorigenic mutation and future therapeutics undoubtedly exist. Indeed, studies of the genomes of parathyroid adenomas using a variety of methods have revealed a complex and heterogeneous landscape of chromosomal and DNA sequence level changes [8-13]. Identification of novel, recurrently altered genes has been difficult due to a low frequency of mutation observed in candidate genes and the limited number of cases examined [8, 9]. For example, *EZH2* mutation was observed in 1 of 8 adenoma exomes, and overall in 2/193 adenomas after direct sequencing of additional samples [8]. Thus, to shed more light on the molecular pathogenesis of this disorder, we subjected a larger cohort consisting of 19 pairs of parathyroid adenoma and matched germline DNA to whole exome sequencing.

## RESULTS

A total of 59 confirmed somatic nonsynonymous mutations were identified in 16 of the 19 adenoma samples, with each tumor harboring 0 to 7 mutations. This corresponds to an average of ~3.7 mutations per sample in agreement with previous parathyroid adenoma exome studies, and is considered to be on the lower spectrum of nonsynonymous mutations in human tumors in general [8, 9, 14]. Two of these 59 somatic mutations (in 2 separate adenoma cases) involved the *MEN1* gene, a previously established contributor to parathyroid neoplasia. Of the genes that were mutated, the overwhelming majority were mutated in only 1 tumor. Only the *ZFX* gene was mutated in more than one tumor and in neighboring codons suggestive of a mutational hotspot prompting further investigation.

**Figure 1:**
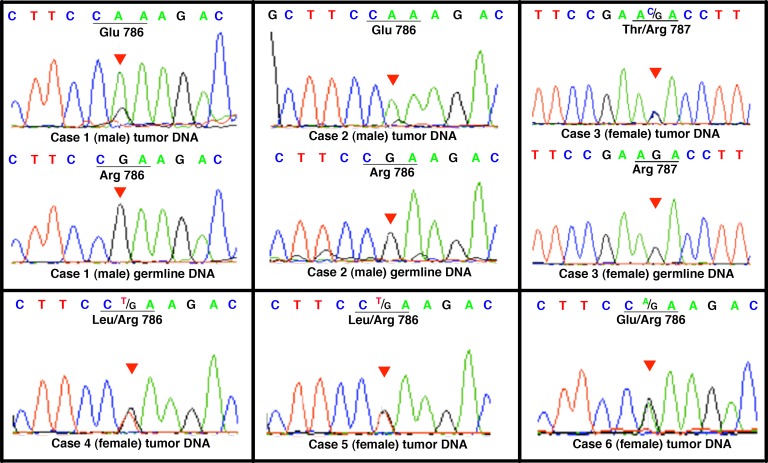
Direct genomic DNA sequencing of *ZFX* R767/768 mutations Chromatograms of the *ZFX* R786/787 mutations in adenoma samples, with matched germline control DNA when available. Germline DNA samples were unavailable for patients 4, 5 and 6. The codons where mutations were identified are underlined for clarity. The red triangle in each chromatogram indicates the location of the mutation.

Somatic mutations in *ZFX* codons 786 and 787 were identified by exome sequencing in two tumor samples. Subsequently, resequencing of the *ZFX* region encompassing these codons in an expanded collection of parathyroid adenomas revealed mutations in 6 of 130 samples (Table 1). In all cases the mutation affected one of the same two codons 786/787, which encode arginine residues that are highly conserved among vertebrates including lamprey, zebrafish, *X. tropicalis*, chicken, elephant, dog, mouse and Rhesus monkey, as per the Multiz Alignment datatrack of the UCSC Genome Browser [15]. The resulting arginine to glutamine, threonine, or leucine substitutions are all predicted by SIFT to cause damaging functional effects to the encoded *ZFX* protein [16]. Sequence analysis of the entire coding region of *ZFX* on 60 parathyroid adenoma samples did not reveal additional mutations beyond those identified in codons 786 and 787. Three of the six mutations were verified as acquired somatic mutations (Figure 1). The somatic status of the other 3 mutations could not be verified due to lack of available germline control materials. However, none of these mutations were registered in dbSNP137 database of genomic variants in the general population, which contains more than 4000 alleles at the *ZFX* genomic locus.

**Table 1:** Summary of recurrent ZFX mutations in sporadic parathyroid adenomas

Case ID	Gender	Mutation annotation*	Somatic/Germline	Genomic position**
1	M	c.2357G>A p.R786Q	somatic	ChrX: 24229432
2	M	c.2357G>A p.R786Q	somatic	ChrX: 24229432
3	F	c.2360G>C p.R787T	somatic	ChrX: 24229435
4	F	c.2357G>T p.R786L	ND***	ChrX: 24229432
5	F	c.2357G>T p.R786L	ND***	ChrX: 24229432
6	F	c.2357G>A p.R786Q	ND***	ChrX: 24229432

* Mutation annotations were based on Refseq ZFX transcript NM_003410.

** Genomic positions were based on human genome reference sequence assembly GRCh37.

*** Somatic vs germline status of mutations identified in tumor cases 4, 5 and 6 was not determinable due to unavailability of matched germline DNA.

In our whole-exome sequencing, samples harboring somatic *ZFX* mutations did not overlap with those containing somatic mutations in *MEN1*, which were detected in 2 of the 19 sporadic adenomas. This *MEN1* intragenic mutation frequency of ~11% is in agreement with earlier literature, which found a range between 12- 20% [11-13], and contrasts with recent suggestions that the frequency might be considerably higher (up to 35-40%) [8, 9, 17-20]. No mutations in genes recently proposed to be implicated in parathyroid adenoma tumorigenesis, such as *CTTNB1*, *EZH2* and *POT1*, were identified, attesting to the rarity of these candidate genes' potential contributions [21].

**Figure 2:**
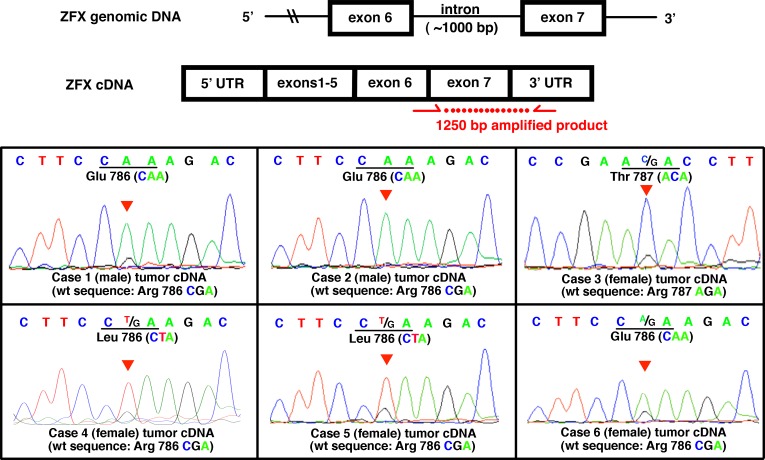
Expression of mutation-bearing *ZFX* alleles in parathyroid adenomas Top: Schema of cDNA amplification to detect mutant transcripts extracted from parathyroid adenomas with *ZFX* mutations. The red arrows indicate the location of the forward and reverse primers. The forward primer spanned the junction of exon 6 and exon 7, which prevented amplification of genomic DNA. Bottom: Sequence chromatograms of *ZFX* cDNA from the parathyroid adenomas with R786/787 mutations. The mutant *ZFX* transcripts were present in each of the mutation-bearing adenoma samples. For each box, from the top, the first line of nucleotide sequence represents base calls from tumor cDNA. Codon and notation of the mutant/prominent allele is labeled beneath the first line. The red triangle indicates the location of the mutant base in the chromatogram. For convenience, the wild-type (standard reference) *ZFX* sequences are labeled in parentheses beneath each chromatogram. Nucleotide letters in the chromatogram and codon labels are color coded to match.

**Figure 3:**
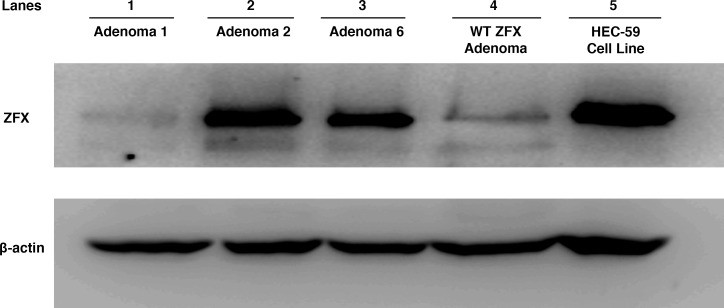
Western blot analysis of *ZFX* expression in parathyroid adenomas Proteins were extracted from three *ZFX* mutation-bearing parathyroid adenomas (cases 1, 2, and 6 in lanes 1, 2, and 3 respectively), one adenoma with wildtype *ZFX* sequence (lane 4) and a human endometrial carcinoma cell line (HEC-59; Lane 5). Western blot analysis was then performed using anti-*ZFX* antibodies. Expression of *ZFX* at the protein level was detected in all adenoma samples, regardless of their mutation-bearing status, as well as the cell line. Imunoblotting for ß-actin served as loading control. HEC-59 cell lysate served as positive control for *ZFX* protein expression. Since only one allele of the *ZFX* gene exists in the male human genome (XY), positive immunoblotting from lanes 1 and 2 suggest the mutant form of *ZFX* is expressed at the protein level.

Sanger sequencing of the RT-PCR product from mRNA extracts of *ZFX* mutation-bearing tumors showed that the mutant *ZFX* alleles were expressed in each case (Figure 2). All tumors samples from female patients showed evidence of expression of both alleles (wild type and mutant) in agreement with prior observations that *ZFX* escapes X-inactivation [22]. In addition, three of the *ZFX* mutation-bearing tumors were available for extraction of protein for western blot analysis. Two of the three samples showed modest to robust expression of *ZFX*; one of these samples is from a male patient and contains only a single (mutant) *ZFX* allele, further documenting that the *ZFX* mutation is expressed (Figure 3).

## DISCUSSION

*ZFX* is a member of the Krueppel C2H2-type zinc finger protein family and is known to have a regulating role in embryonic stem cell renewal [23]. The encoded protein product includes 13 zinc finger domains [22]. The mutated residues identified in our series of parathyroid neoplasms are located in the zinc finger subdomain that may directly interact with DNA. Since DNA is negatively charged and the *ZFX* mutations uniformly altered a positively charged arginine to a non-charged residue, they appear likely to have resulted in abnormalities in *ZFX*'s DNA binding affinity and/or specificity. In addition, the mutations are located approximately 20 amino acids upstream from the C-terminus, suggesting that the zinc finger domain affected by the mutations might be located at the surface of the protein and may be involved in inter-molecular interactions (such as protein to DNA) as opposed to being an internal structural component of the protein.

Although genomic hybridization and loss of heterozygosity (LOH) studies have suggested the presence of an undiscovered parathyroid tumor suppressor gene on the X chromosome [11, 24, 25], and notwithstanding that *ZFX* deletion has led to tumorigenesis in an experimental liver cancer model in mice [26], it does not appear that *ZFX* is acting as a classic two-hit human tumor suppressor in parathyroid adenomas. In particular, we observed no mutations that would be expected to inactivate the *ZFX* gene product, such as early stop codons or frameshifts; in addition, none of the female patients' tumor samples that harbored a *ZFX* mutation showed LOH at the corresponding wild type locus.

In contrast, the striking specificity of the genetic alterations we observed in R786/787 supports the idea that mutant *ZFX* is a direct-acting oncogene in the context of parathyroid adenomas, and suggests that only this exceedingly limited range of *ZFX* mutations is able to provide a crucial gain of function, or introduce a new function, needed to confer a selective advantage on a parathyroid tumor progenitor cell. Intriguingly, *ZFX* was recently revealed to be a transcriptional target of cyclin D1 [27], linking *ZFX* to an established oncogene product and oncogenic pathway in parathyroid and other cell types. Further, knockdown of *ZFX* has been reported to inhibit cellular proliferation in human laryngeal squamous cell carcinoma, gastric cancer, malignant glioma, non-small cell lung carcinoma and prostate cancer [28-32]. Finally, a solitary *ZFX* R786Q somatic mutation in endometrial carcinoma has been registered in the COSMIC database (endometrial cancer; mutation ID: COSM1119462). Until now, pathogenetic importance could not be attributed to this isolated finding, but in the context of our current observations, the listing of this mutation in a different tumor type suggests that recurrent R786/787 mutations in *ZFX* may contribute to human tumorigenesis in a broader fashion.

This study provides the first genetic evidence of recurrent *ZFX* mutations in human neoplasia. The mechanisms by which *ZFX* mutations contribute to a selective growth advantage in parathyroid, and potentially other types of tumor cells, and whether *ZFX* is involved in cancer stem cell renewal, remain to be elucidated. Further, these observations highlight the mutated zinc-finger domain as a new focal point for understanding *ZFX*'s normal and tumorigenic functions, and for development of molecular-targeted therapeutics.

## METHODS

### Patients and Samples

Tumor samples were obtained from patients who had undergone parathyroidectomy for management of primary hyperparathyroidism with typical presentations, histopathologically and surgically proven to be caused by a single sporadic parathyroid adenoma with no atypical and/or malignant features. All patients had a negative family history of primary hyperparathyroidism, a negative personal or family history suggestive of multiple endocrine neoplasia, and a negative history of head and neck irradiation. For most patients, blood samples were obtained to serve as a source of matched germline DNA when available. DNA was extracted from parathyroid adenoma specimens by proteinase K digestion followed by phenol-chloroform extraction and ethanol precipitation. Selection of tumor samples for exome sequencing required availability of both tumor and matched germline control DNA for a given patient. All samples were obtained with informed consent in accordance with institutional review board-approved protocols.

### PCR and Sanger Sequencing

Primers were designed with Primer3Plus [33]. Primers utilized are listed in Appendix Table 1. PCR reactions were performed in 20 ul reaction columns containing 25 ng of sample DNA, 200 uM of dNTP, 2 mM magnesium chloride, and 1.25 U of Gold DNA polymerase (Applied Biosystems, Foster City, CA, USA). The thermal cycles began with a single denaturation step of 95°C for 10 minutes; 35 cycles of 95°C for 30s, 58°C for 1 min, and 72°C for 30s; and ended with a single extension step at 72°C for 10 minutes. The PCR products were purified with ExoSAP-IT (Affymetrix, Santa Clara, CA) and were sequenced using standard Sanger sequencing methodology (GeneWiz Inc., South Plainfield, NJ). Sequence data were analyzed using the Staden Package to align multiple tumor sample sequences along with reference sequences and to visually scrutinize for possible variants in the tumor and control samples [34]. Potential mutations were confirmed by resequencing from independent PCR reactions.

### cDNA Generation/ Transcript Detection

cDNA libraries were generated from tumor RNA extracted from frozen samples using Qiagen RNeasy Mini Kit (Ambion Inc. Austin, Texas). The samples were then reverse transcribed using High Capacity cDNA Reverse Transcription Kit (Applied Biosystems, Foster City, California) to generate cDNA libraries. Subsequent PCR reactions were performed with a primer set that target-amplified a portion of exon 6 and the entire region of exon 7 of the *ZFX* gene to detect the transcripts of *ZFX* mutant alleles (Figure 2 and Appendix Table 1). Amplified cDNA products were Sanger sequenced and the sequence data were aligned back to the genome to confirm the amplified product is specific and from the *ZFX* gene transcript.

### Protein extraction

Roughly 50 mg of frozen tumor tissue was minced, homogenized and sonicated in 150 μL RIPA buffer supplemented with proteinase inhibitors. The samples were incubated for 30 minutes at 4°C and centrifuged at 13,000 g for 15 minutes at 4°C. Supernatants were collected for western blot analysis using *ZFX* antibodies (Cell Signaling Technology Inc., Danvers, MA). Protein extracted from human endometrial carcinoma cell line (HEC-59) was used as a positive control based on known *ZFX* expression in human endometrial carcinoma cell lines [35].

### Exome sequencing and data analysis

High molecular weight DNA was used to generate libraries for sequencing following Illumina recommended procedures (San Diego, CA). Extracted DNA was fragmented using Covaris Adaptive Focused Acoustic system or a nebulizer shearing system. The resulting DNA fragments were subjected to end repair leaving a 5′-A overhang on both ends. Subsequently, an adaptor was ligated to the overhanging A; these adaptor molecules allow for subsequent priming for amplification and sequencing read out. A PCR purification step was then followed to add additional sequences and increase library concentration. Post PCR, the library was subjected to target enrichment using Agilent's SureSelect Whole Exome kit protocol (Danbury, CT), which uses magnetic bead based RNA-baits designed to capture DNA fragments that contain sequences from the human coding region only. After target enrichment and purification, the libraries were sequenced on the Illumina Genome Analyzer II or HiSeq2000.

Sequence raw data were aligned with BWA alignment software [36] to NCBI human genome build GRCh37 and variants were called with SAMTOOLS, Dindel and Genome Analysis Toolkit [37-39]. After these steps, the exome data were subsequently analyzed and annotated with in-house software to identify potential somatic mutations, i.e. those present in the tumor but not in the matched germline exome. All potential somatic mutations identified through exome analysis were confirmed via targeted PCR and Sanger sequencing.

## SUPPLEMENTARY TABLE


