# Recurrent ZFX mutations in human sporadic parathyroid adenomas

**Published:** 2014-05-06

**Authors:** Chen-Pang Soong, Andrew Arnold

Eratum: Recurrent ZFX mutations in human sporadic parathyroid adenomas.

Chen-Pang Soong, Andrew Arnold

(2014).Oncoscience 1: 360-366.

***PMCID***: PMC4278311. PMID: 25594030

In Figures 1 and 2, the 3-letter amino acid code labels Glu should have been Gln. The corrected Figures 1 and 2 are provided here.

**Figure 1:**
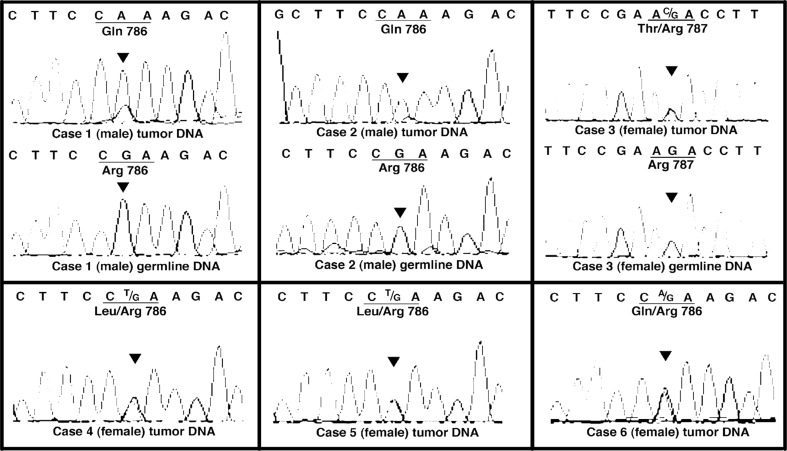
Direct genomic DNA sequencing of ZFX R767/768 mutations Chromatograms of the ZFX R786/787 mutations in adenoma samples, with matched germline control DNA when available. Germline DNA samples were unavailable for patients 4, 5 and 6. The codons where mutations were identified are underlined for clarity. The red triangle in each chromatogram indicates the location of the mutation.

**Figure 2:**
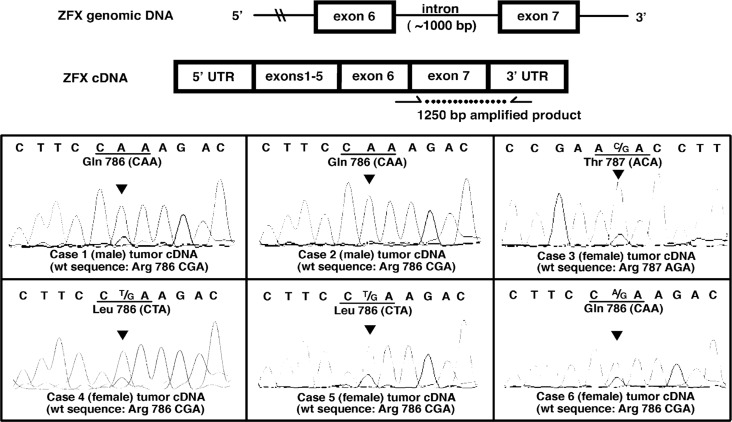
Expression of mutation-bearing ZFX alleles in parathyroid adenomas **Top: Schema of cDNA amplification to detect mutant transcripts extracted from parathyroid adenomas with ZFX mutations.** The red arrows indicate the location of the forward and reverse primers. The forward primer spanned the junction of exon 6 and exon 7, which prevented amplification of genomic DNA. **Bottom: Sequence chromatograms of ZFX cDNA from the parathyroid adenomas with R786/787 mutations.** The mutant ZFX transcripts were present in each of the mutation-bearing adenoma samples. For each box, from the top, the first line of nucleotide sequence represents base calls from tumor cDNA. Codon and notation of the mutant/prominent allele is labeled beneath the first line. The red triangle indicates the location of the mutant base in the chromatogram. For convenience, the wild-type (standard reference) ZFX sequences are labeled in parentheses beneath each chromatogram. Nucleotide letters in the chromatogram and codon labels are color coded to match.

