# Supplementary material for: Recurrent ZFX mutations in human sporadic parathyroid adenomas
Source: Oncoscience. 2014 May 6;1(5):360–6. doi: 10.18632/oncoscience.116 (PMC4278311; doi:10.18632/oncoscience.116)
Supplement: Supplementary file 1 [file oncoscience-01-0360-s001.pdf]

Appendix Table 1. Primer sequences

A. Primer set for *ZFX* RT-PCR reactions; annealing temperature 58°C. Primer sequences are shown 5’ to 3’.

Primer set	Forward	Reverse
1	CAGGCAGTACCAAACAGCAA	TTTTCTGCTTCAAGGCCAAT

B. *ZFX* resequencing primers; annealing temperature 58°C. Primer sequences are shown 5’ to 3’.

ZFX	Forward	Reverse
CDS1	TCATGATGGCATTTAATACCTG	GCAATTCATTGATGACAACCA
CDS2a	GCTAATCTGTTTTCCCAGTAGAAA	CACATCTGCTTCTTCCATGA
CDS2b	CGTTGTTATAGAAGATGTTCAGTGC	TCAGTGACAATTTCTGCTTCCA
CDS2c	ACGTCTTGACGGGTGATTCT	AGGCTGCAAATGGAAACAAC
CDS3	GCCTGGGTGACAGTGAGACT	CAGAAGAGACTCCAAATGTTCTCA
CDS4	AAAAAGAAATAGGTGGAAATGCAG	TTCAAACACTGGCCACTTAAAA
CDS5	AAACGTGTTTCCTGTGATCTCT	CCACAGAAACCATTTTTCAATC
CDS6	TTTTCGTTGTTGTAGTTAATGAAGAA	TGTGGATACATACTAGAGTTTCCTCTG
CDS7a	TTCGCAAAGAAACTGGAACA	CATTCAATGGCCTTCTCTGC
CDS7b	CATGAAAAACCATCCCGAAC	TGGCGATTCAATAACCCTTG
CDS7c	TGCAGGGGCTTTGTTTACTC	TGCTTAGTTTTGACATGCGTTT
CDS7d	CATACTGGGGAGAAGCCGTA	CATGTCACACTTATGGGGGTAG
CDS7e	CATTGCGACCACAAGAGTTC	GCTCACTCTGTTGCCTAAATCC
CDS7f	GCAGATCCATTTGTTCTAAGTCG	TGTCGCATTATGTGCTGGTT
CDS7g	ACGGCACGTTATTTCCATTC	TGAATGAGACTGATTGGCTTT
